# Comparison of different intervention methods to reduce the incidence of venous thromboembolism: study protocol for a cluster-randomized, crossover trial

**DOI:** 10.1186/s13063-023-07868-y

**Published:** 2023-12-19

**Authors:** Qin Tan, Lu Zhou, Yihan Lu, Weifeng Huang

**Affiliations:** 1 Department of Critical Care Medicine, Shanghai Sixth People’s Hospital Affiliated to Shanghai Jiao Tong University School of Medicine, 600 Yishan Road, Shanghai, 200233 China; 2 Department of Epidemiology, School of Public Health, Fudan University, Shanghai, China; 3 National Health Commission Key Laboratory of Health Technology Assessment (Fudan University), 131 Dong’ an Road, Shanghai, 200032 China

**Keywords:** Venous thromboembolism, Orthopedic trauma patients, Low molecular weight heparin, Cluster-randomized crossover

## Abstract

**Background:**

Venous thromboembolism (VTE) remains a priority challenge among orthopedic trauma patients. It is crucial to further improve the prophylaxis against VTE in routine orthopedic treatment. This study aims to compare the efficacy of two low molecular weight heparin (LMWH) regimens and additional intermittent pneumatic compression in preventing VTE among orthopedic trauma patients.

**Methods and analysis:**

This is a cluster-randomized crossover clinical study conducted in four hospitals in Shanghai from December 2019 to December 2023. The unit of randomization is orthopedic wards, and each ward will define a cluster. All clusters will implement four diverse intervention measures and one control measure in a given random sequence. Perioperative orthopedic trauma patients aged ≥ 18 years with stable vital signs, Caprini score > 2, and no contraindication of anticoagulation or intermittent pneumatic compression (IPC) devices will be eligible. The sample size will be determined to be 2590, considering cluster effect, period effect, and interactions. We will generally use the intention-to-treat (ITT) at the subject level for each outcome. For the primary outcome of the study, the incidence of VTE will be presented as risk ratio and 95% CIs. Generalized estimating equation (GEE) will be deployed to compare differences and adjust cluster effect, period effect, and interaction among interventions and periods if applicable.

**Discussion:**

VTE is a complication that cannot be underestimated after major orthopedic surgery. Early identification, early assessment, and early prevention can significantly reduce the incidence of VTE. Most guidelines recommend both medical and physical prevention, and we hope to demonstrate how they would affect the incidence among perioperative orthopedic patients. We want to explore if there is a difference between the two types of LWMH with or without an IPC device to provide more evidence for future guidelines and prevent more patients from the threat of VTE.

**Ethics and dissemination:**

The study received approval from the IRB of the coordinating center and all participating hospitals. Findings will be disseminated through peer-reviewed publications and conference presentations.

**Trial registration:**

ChiCTR1900027659. Registered on 17 November 2019

**Supplementary Information:**

The online version contains supplementary material available at 10.1186/s13063-023-07868-y.

## Background

Venous thromboembolism (VTE) refers to the abnormal coagulation of blood in the vein, causing complete or incomplete occlusion of blood vessels, which belongs to venous reflux disorder disease [1]. It has been documented that 40–60% of surgical or medical patients remained at risk of VTE, ranking as the third most common cardiovascular disease following heart attack and cerebral infarction [2, 3]. However, VTE prophylaxis remains underused. The ENDORSE study found that only 40% of at-risk medical patients received VTE prophylaxis in 32 countries [4]. Recently, our retrospective cohort study confirmed that approximately 60% of orthopedic trauma patients received VTE prophylaxis, which was considerably higher compared to previous studies. However, VTE incidence was determined to be 19% [5], similar to that among the orthopedic trauma patients without receiving prophylaxis in Canada [6]. This finding raised a concern in the effectiveness of VTE prophylaxis.

It is routinely recommended initiating low molecular weight heparin enoxaparin (LMWH) as pharmacological prophylaxis for orthopedic trauma patients [7]. Multiple studies focused on monitoring plasma anti-Xa levels to adjust the dosage of LMWH administered to orthopedic trauma patients [7]. It suggested that the best dosage would be achieved when administering at least the third dose of LMWH [8]. Compared with a fixed dosage, a weight-based dosage may be more effective in reaching the expected anti-Xa level [9]. Additionally, weight has been proven to be a key predictor of anti-Xa levels [10]. Thus, our study is designed to administer a weight-based dosage of LMWH, instead of a fixed dosage as usual, to achieve a better effect in preventing VTE. Our retrospective cohort study found that varying dosage as well as diverse LMWH regimens had different prophylaxis effects, as described elsewhere [11]. Moreover, intermittent pneumatic compression (IPC) is used as mechanical prophylaxis when patients have a contraindication to pharmacological prophylaxis [12, 13]. However, previous studies have been limited by non-randomized design, small sample size, and negative findings, when recommending IPC for orthopedic trauma patients [14, 15]. Furthermore, whether the combination of IPC and LMWH may reduce the risk of VTE remains uncertain [16]. Therefore, we decided to design and perform a cluster-randomized crossover trial to further determine the efficacy of multiple interventions in preventing VTE among orthopedic trauma patients.

### Hypothesis and outcomes

The hypothesis is that the combination of LMWH and IPC can reduce the risk of VTE. The primary outcome of this study is the incidence of VTE, defined as on VTE observed before the orthopedic surgery, while any form of VTE identified after the surgery. The secondary outcomes included the amount of bleeding during operation and mortality within 30 days.

## Methods and analysis

### Study design

This study is a cluster-randomized crossover, multicenter exploratory trial that is designed to assess the safety and efficacy of multiple intervention measures for preventing VTE among perioperative orthopedic trauma patients. The unit of randomization is the orthopedic wards, and each ward will define a cluster, as the proposed interventions are designed to be implemented in all orthopedic wards rather than in selected individual subjects. The study will be conducted at four hospitals in Shanghai from December 2019 through December 2023, including (1) Shanghai Sixth People’s Hospital, Shanghai Jiao Tong University School of Medicine; (2) Jinshan Branch of Shanghai Sixth People’s Hospital, Shanghai Jiao Tong University School of Medicine; (3) Lingang Compound of Shanghai Sixth People’s Hospital, Shanghai Jiao Tong University School of Medicine; and (4) Shanghai Tenth People’s Hospital, Tongji University. A total of five clusters will be determined in the study, according to the routine amount of orthopedic trauma patients visited in those hospitals.

### Inclusion criteria

Perioperative orthopedic trauma patients are considered eligible when they meet the following criteria: aged ≥ 18 years with stable vital signs, Caprini score > 2, and no contraindication of anticoagulation or IPC devices during the study period. Furthermore, patients will be excluded when they have severe open injury in lower limbs which cannot tolerate ultrasonography, venous thromboembolism at enrollment, hepatic and/or renal dysfunctions, and hemorrhage or high risk of hemorrhage in the closed cavity. Female patients in pregnancy or lactation will be excluded as well.

### Interventions

A total of four intervention measures and one control measure will be implemented as follows.

Intervention 1: Fraxiparine (Glaxo Smith Kline, England) nadroparin calcium will be given once a day subcutaneously, with the dosage of 100 Axa IU/kg taking patient’s body weight into account.

Intervention 2: Clexane (Sanofi, France) enoxaparin sodium will be given once a day subcutaneously, with the dosage of 100 Axa IU/kg taking patient’s body weight into account.

Intervention 3: Fraxiparine (Glaxo Smith Kline, England) nadroparin calcium will be given once a day subcutaneously, with the dosage of 100 Axa IU/kg taking patient’s body weight into account. In addition, IPC (Wonjin Mulsan, China) will be administered twice a day, 20 min each time.

Intervention 4: Clexane (Sanofi, France) enoxaparin sodium will be given once a day subcutaneously, with the dosage of 100 Axa IU/kg taking patient’s body weight into account. In addition, IPC (Wonjin Mulsan, China) will be administered twice a day, 20 min each time.

Control: There is no predetermined intervention. The prophylaxis measure against VTE will be administered based on the orthopedists’ decision for the best of patients. The details of the measures will be recorded.

**Fig. 1:**
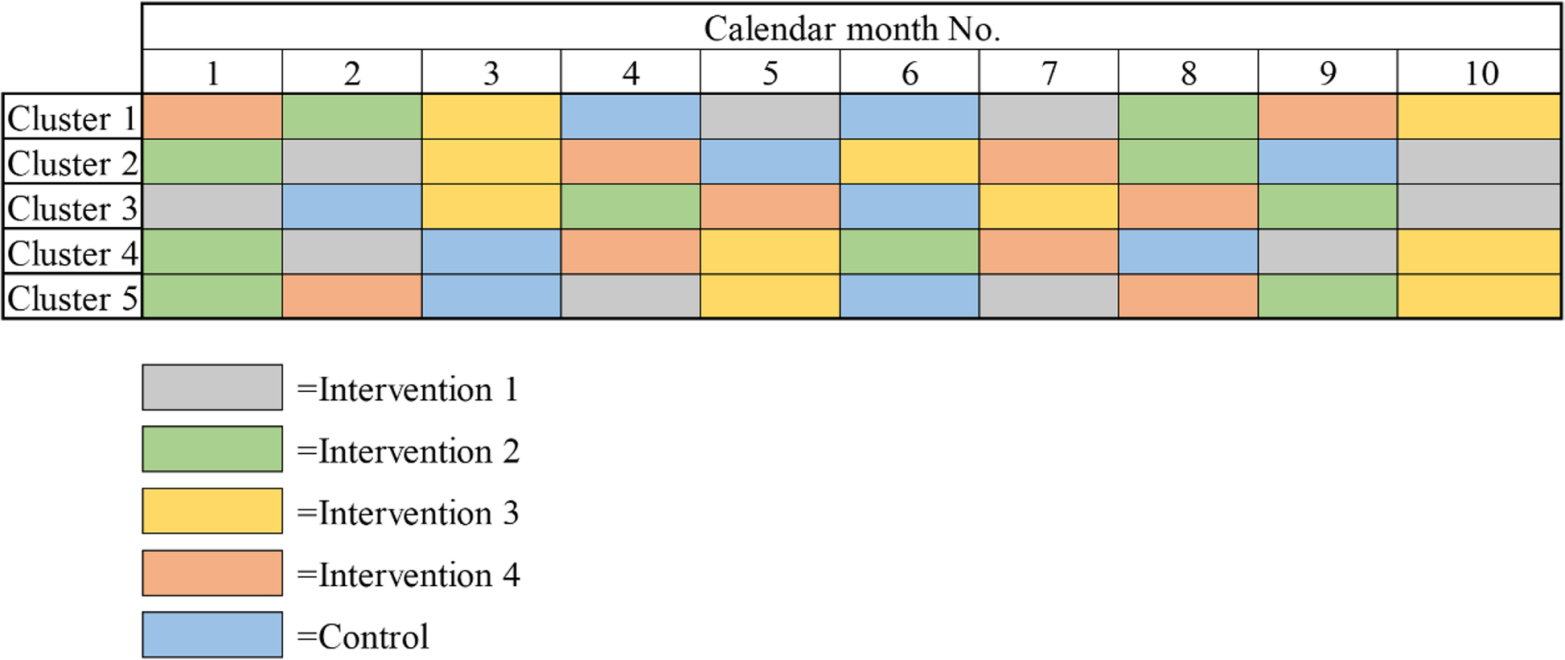
Diagram of the study design. Four diverse intervention measures and one control measure were presented based on ten hypothetical periods of 10 months per cluster

These intervention measures and control measure will alternate along with calendar months (Fig. 1), which means orthopedic trauma patients enrolled in the study will be administered with same measures per cluster per calendar month.

### Proposed sample size

We determined the sample size based on the cumulative incidence of VTE. It is estimated that at least 398 orthopedic trauma patients need to be enrolled in each of the five clusters to achieve a power value of 85% to detect an absolute difference of 10% in the cumulative incidence of VTE between the patients with intervention measures and those with control measures (assuming a VTE incidence of 15% according to our retrospective cohort study [5]) at a two-sided alpha level of 0.05. Consequently, approximately 40 patients will be enrolled per intervention measure/control measure per calendar month in each cluster, totaling 1990 patients. The intraclass correlation coefficient (ICC) was calculated based on two levels, 0.228 for patients within the same cluster/time period and 0.15 for patients within the same cluster/different time periods. Due to the lack of data on ICCs related to the current study design and primary outcome, we chose to use a conservative ICC value in our sample size estimation. Moreover, considering a 20% loss of participants, we determine to enroll a total of 2490 patients in the trial, with 498 in each cluster.

### Randomization and allocation

In this study, five clusters will equally share the proposed sample size. In the implementation, each cluster receives several sequences of intervention periods (including four diverse intervention measures) and control periods, which is determined by a randomization, to achieve the allocated sample size (Fig. 1).

Additionally, orthopedic trauma patients are randomly included in these clusters, which might result in a slightly different number of enrolled participants over the periods across the clusters. Thus, we define the duration of a calendar month for each intervention or control period in these clusters, which may ensure the clusters achieve the sample size within the identical number of sequences.

### Data collection

In this study, the primary outcome is the incidence of VTE, which will be measured by trained orthopedists using ultrasound examination (Mindray M8, 8.0–11.0 MHz, China) [17]. Additionally, adverse events following the administration of LWMH and/or IPC will also be recorded and judged by experienced orthopedists, pharmacists, and intensivists, to assess the safety of multiple intervention measures.

The secondary outcomes include the amount of bleeding during operation and mortality within 30 days. Related data will be collected through the hospital information system (HIS) using a standardized case report form (CRF) and recorded using EpiData 4.2. Participants will be followed up from study enrollment to hospital discharge, 30 days after discharge.

### Quality assurance and data management

Multiple measures will be taken to optimize the implementation of the research activities and ensure the quality of collected data. Before the study initiation, an investigator meeting will be organized, including the principal investigator, orthopedists and senior nurses from the participating orthopedic wards, statisticians, and research assistants. During the meeting, investigators will receive training about study design, implementation plan, and data collection and analysis for all intervention measures. In each orthopedic ward, an experienced staff (A) will be selected and trained about the key aspects of implementation details and further be responsible for monitoring and evaluation. Moreover, all the orthopedic wards will have a learning period within the first 10 days of interventions. Additionally, the measurements used in the study, such as the Caprini score, trauma index score, and VTE assessment, will be recorded [18]. EpiData 4.2 will be used to collect the trial data. In each ward, a trained staff (B) will collect data from HIS using standard CRF. Data cleansing will be conducted by another trained staff (C) every week to ensure the data check and management.

The SPIRIT reporting guidelines were used to ensure the completion of the study protocol (Additional file 1) [19].

### Data analysis

#### Statistical analysis principles

We will generally perform the analysis using the intention-to-treat (ITT) at the subject level for each outcome. All the participants with a recorded outcome will be included in the analysis according to the intervention group to which they have been randomized. Additionally, we will take the cluster-randomized crossover design effect into consideration. Adjustment for multiple comparisons among interventions will be used. The SAS 9.4 software will be used for all statistical analyses.

#### Handling of missing data

The least data missing will be expected. All-important observations will be collected and assessed by trained investigators. At the cluster level, orthopedic wards with incomplete patient recruitment will be included in the primary analysis of study outcomes, considering all participants available in each cluster. To assess the risk of bias related to orthopedic wards that have not completed patient recruitment, multiple imputations will be performed for the primary outcome and presented as sensitivity analyses. At the subject level, when a variable is missing, we will assume that most likely the missing variable has a normal or mean value.

### Subject flow

**Fig. 2:**
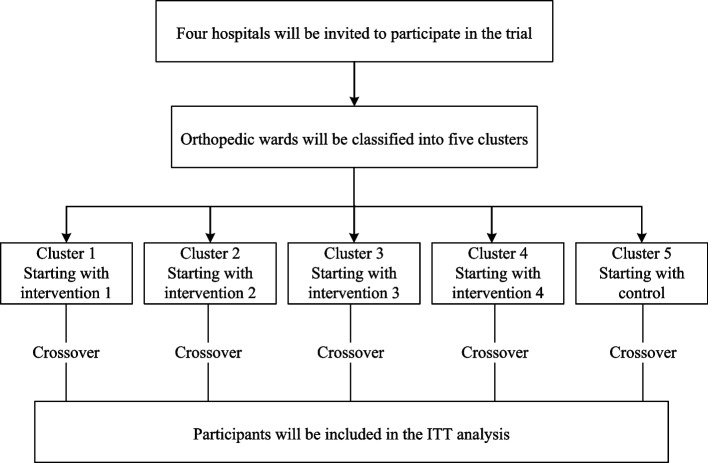
Study flow diagram. *ITT*, intention-to-treat

Figure 2 displays the flow of the trial.

### Primary analysis

For the primary outcome of the study, the incidence of VTE will be presented as risk ratio and 95% CIs. Generalized estimating equation (GEE) will be deployed to compare differences and adjust cluster effect, period effect, and interaction among interventions and periods if applicable.

### Secondary analysis

For the secondary outcomes, the amount of bleeding during operation and mortality within 30 days will be presented as mean (or median) difference and 95% CI. GEE will be deployed to compare the difference among interventions or clusters. All the analyses will adjust cluster effect, period effect, and interaction among interventions and periods if applicable.

### Subgroup analysis

For the primary outcome, efficacy among interventions will be determined according to the Caprini score and TI score, as well as sociodemographic and clinical groups. To test for interactions, the consistency of intervention effect across the subgroups will be assessed. Bonferroni correction will be used to adjust subgroup analyses for multiple comparisons.

## Discussion

VTE is a complication that cannot be underestimated after major orthopedic surgery, which is challenging to find and diagnose. Early identification, early assessment, and early prevention can significantly reduce the incidence of VTE. Numerous approaches to prevent VTE have been developed and evaluated. Most guidelines recommend both medical and physical prevention, and we hope to demonstrate how they would affect the incidence among perioperative orthopedic patients.

Our study design has strengths. First, the design of a clustered crossover trial would facilitate the statistical power to detect possible differences among multiple intervention measures. Second, the format of orthopedic wards as randomized clusters will enhance the fidelity of the interventions, which will be performed by same orthopedists. Third, the study will include a larger sample size of orthopedic trauma patients, which will improve the robustness of the findings. However, there is a limitation to this study design. The crossover design might lead to difficulty in the implementation of the trial.

### Trial status

The protocol version number: 001; date: 17 November 2019.

**Fig. 3:**
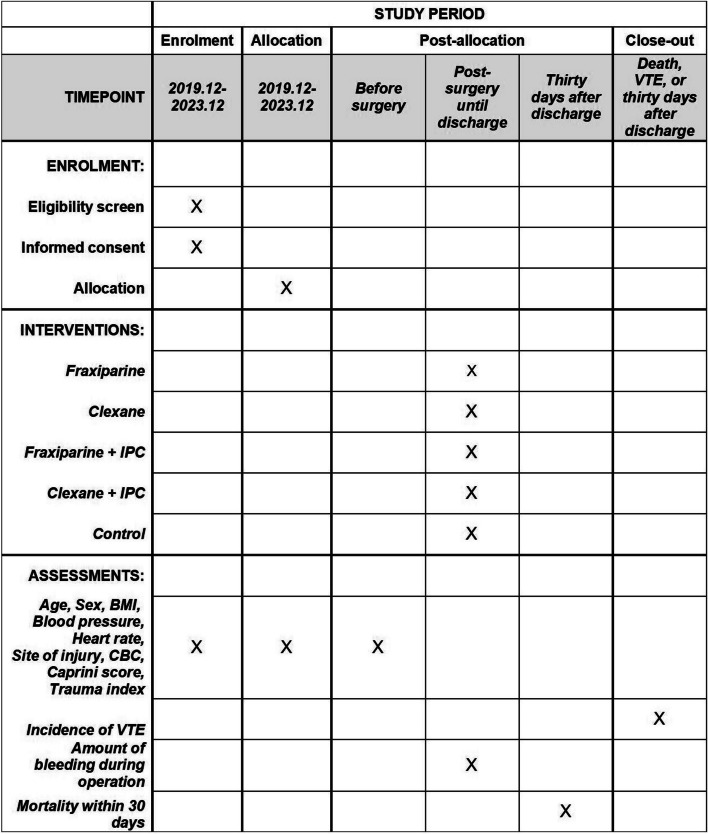
The enrollment, intervention, and assessment schedule for this study

Recruitment began in December 2019 and is expected to complete in December 2023 (Fig. 3).

The date of the latest Ethical Approval Extension document is December 21, 2022, with a validity period of 1 year, which is still within the current effective period. If necessary, we will apply for a new extension.

### Supplementary Information

**Additional file 1:.** SPIRIT checklist.

## Data Availability

The datasets generated during and/or analyzed during the current study are not publicly available due to the privacy policy but are available from the corresponding author upon reasonable request.

## Abbreviations

VTE: Venous thromboembolism
LMWH: Low molecular weight heparin
IPC: Intermittent pneumatic compression
ITT: Intention-to-treat
GEE: Generalized estimating equation
HIS: Hospital information system
CRF: Case report form
